# Identification and Evaluation of New Potential Inhibitors of Human Neuraminidase 1 Extracted from *Olyra latifolia* L.: A Preliminary Study

**DOI:** 10.3390/biomedicines9040411

**Published:** 2021-04-11

**Authors:** Camille Albrecht, Zachée Louis Evariste Akissi, Philomène Akoua Yao-Kouassi, Abdulmagid Alabdul Magid, Pascal Maurice, Laurent Duca, Laurence Voutquenne-Nazabadioko, Amar Bennasroune

**Affiliations:** 1 UMR 7369, Matrice Extracellulaire et Dynamique Cellulaire (MEDyC), UFR Sciences Exactes et Naturelles, CNRS, Université de Reims Champagne-Ardenne, 51097 Reims, France; camille.albrecht@univ-reims.fr (C.A.); pascal.maurice@univ-reims.fr (P.M.); laurent.duca@univ-reims.fr (L.D.); 2 UMR 7312, Institut de Chimie Moléculaire de Reims (ICMR), UFR Sciences Exactes et Naturelles, CNRS, Université de Reims Champagne-Ardenne, 51097 Reims, France; zachee-louis-evariste.akissi@univ-reims.fr (Z.L.E.A.); abdulmagid.alabdul-magid@univ-reims.fr (A.A.M.); 3 Laboratoire de Constitution et Réaction de la Matière, UFR Sciences des Structures de la Matière et de Technologie, Université Félix Houphouët-Boigny, 22 BP 582 Abidjan, Cote D’Ivoire; kouassiap@yahoo.fr

**Keywords:** *Olyra latifolia*, natural bioactive molecules, Elastin Receptor Complex, neuraminidase 1, sialidase activity

## Abstract

Sialidases, also called neuraminidases, are involved in several human pathologies such as neurodegenerative disorders, cancers, as well as infectious and cardiovascular diseases. Several studies have shown that neuraminidases, such as neuraminidase 1 (NEU-1), may be promising pharmacological targets. Therefore, the discovery of new selective inhibitors of NEU-1 are necessary to better understand the biological functions of this sialidase. In the present study, we describe the isolation and characterization of nine known compounds from *Olyra latifolia* L. leaves. This plant, known to have several therapeutic properties, belongs to the family of Poaceae and is found in the neotropics and in tropical Africa and Madagascar. Among the purified compounds, feddeiketone B, 2,3-dihydroxy-1-(4-hydroxy-3,5-diméthoxyphényl)-l-propanone, and syringylglycerol were shown to present structural analogy with DANA, and their effects on membrane NEU-1 sialidase activity were evaluated. Our results show that they possess inhibitory effects against NEU-1-mediated sialidase activity at the plasma membrane. In conclusion, we identified new natural bioactive molecules extracted from *Olyra latifolia* as inhibitors of human NEU-1 of strong interest to elucidate the biological functions of this sialidase and to target this protein involved in several pathophysiological contexts.

## 1. Introduction

Neuraminidase 1 (NEU-1) is an exoglycosidase which removes terminal sialic acid residues from glycoproteins, glycolipids, and oligosaccharides. Sialidases are widely distributed among species [1]. NEU-1 is implicated in the appearance and progress of various diseases notably through its ability to act as an elastin degradation sensor and to transmit elastin-derived peptides (EDP) signaling [2]. For example, NEU-1 activity is implicated in the development of diabetes due to an enhancement of insulin resistance through insulin receptor desialylation [3]. Interestingly, NEU-1 and the underlying activation of the PI3Kγ signaling pathway promote atherosclerosis onset in mice [4]. Otherwise, NEU-1 is associated with immune thrombocytopenia [5] and EDP contribute also to the development of nonalcoholic steatohepatitis [6]. Furthermore, this enzyme is implicated in the development of various cancers such as melanoma [7,8,9], breast [10,11,12,13], and ovarian [14] cancers.

At present, there are no commercially available inhibitors that are selective for NEU-1, especially due to the lack of structural data. Indeed, only the crystallographic structure of NEU-2 was determined [15]. NEUs are assumed to share a common β-propeller structure organized in six blades with highly conserved motifs implicated in their catalytic activity [16]. The role of NEU-1 is evaluated mainly using the broad-spectrum sialidase inhibitor DANA (N-Acetyl-2,3-dehydro-2-deoxyneuraminic acid) or inhibitors of bacterial or viral NEUs, such as zanamivir or oseltamivir. Among them, Tamiflu, with its active metabolite oseltamivir phosphate, is able to reduce NEU-1 sialidase activity [13,17]. DANA is currently used as a sialidase inhibitor blocking the Elastin Receptor Complex (ERC)-related signaling [3,4,18]. The ERC is a membrane heterotrimeric complex composed of three subunits, the elastin-binding protein which binds elastin-derived peptides (EDP) and tropoelastin, Protective Protein/Cathepsin A (PPCA) that ensures the integrity of the ERC, and the catalytic NEU-1 subunit [19,20,21]. Only two selective inhibitors specific of human NEU-1, the C5-hexanamido-C9-actetamido-DANA [22] and the C9-amido analogue of DANA [23], have been identified. Recently, a promising strategy has been developed using transmembrane peptides to inhibit dimerization and the activation of NEU-1 [24,25]. Thus, identifying new compounds with selective inhibition activity against NEU-1 remains a very exciting challenge.

*Olyra latifolia* L. is a cane-like grass, thin woody culms which can be up to 4 m erected, more or less scandent, from a brief rhizome, and belongs to the family of Poaceae. *O. latifolia* is distributed in the neotropics, from the Southeast of the United States, passing through Central America to the Caribbean in South America and is also found in tropical Africa and Madagascar [26]. This plant is known to have several therapeutic properties. Indeed, in the Central African Republic, the root decoction of *O. latifolia* is used in the treatment of female infertility and orchitis [27]. In Ivory Coast, the leaves are used for treating diabetes and the diabetic wound [28]. The leaves and roots are used in Eastern Nicaragua for the treatment of infections, rashes, and sores [29]. In Guyana, the leaves are macerated and then applied to the cut umbilical cord of babies to prevent infections, whereas the maceration of the flowers is used as a head bath to get rid of dandruff [30]. Previous phytochemical studies have identified the presence of carbohydrates such as fructans, phenolic acids such as caffeic acid, ferulic acid, gentisic acid, *p*-coumaric acid, and *p*-hydroxy-benzoic acid, flavonoids such as flavones and flavonols, anthocyanins, and leucoanthocyanins [31]. Similarly, phytochemical screening carried out on the aqueous extract of the leaves has shown the presence of alkaloids [29].

The aim of the present study was to identify natural compounds extracted from *Olyra latifolia* having structural analogy with DANA and to evaluate their effects on human NEU-1 sialidase activity. Indeed, as the leaves of this plant are used to treat several pathologies such as diabetes, the present work could allow to isolate some active principles targeting NEU-1 and responsible of their medicinal activity. In the present paper, we describe the isolation and characterization of nine known compounds, identified by comparing their spectroscopic data with those reported in the literature. Among the extracted compounds, feddeiketone B, 2,3-dihydroxy-1-(4-hydroxy-3,5-diméthoxyphényl)-l-propanone, and syringylglycerol were shown to present structural analogy with DANA and to possess inhibitory effects against NEU-1-mediated sialidase activity.

## 2. Materials and Methods

### 2.1. General Procedures

NMR spectra were recorded in CD_3_OD on a Bruker Avance III 500 spectrometer (Bruker, Karlsruhe, Germany). HR-ESI-MS analysis was conducted using a Micromass Q-TOF micro instrument (Micromass, Manchester, UK). Vacuum Liquid Chromatography (VLC) was carried out on Lichroprep RP-C18 (40–63 µm) Merck using a sintered glass No. 4 and vaccum for the elution. Flash chromatography was carried out on a Grace Reveleris system equipped with dual UV and ELSD detection using Grace^®^ cartridges (Silica gel or RP-C_18_) (Grace, Epernon, France). HPLC separations were performed on a Dionex apparatus equipped with an ASI-100 autosampler, an Ultimate 3000 pump, a STH 585 column oven, a diode array detector UVD 340S, and a Chromeleon software. A prepacked RP-C_18_ column (Phenomenex 250 × 10 mm, Luna 5 µ) was used for semi-preparative HPLC. The eluting mobile phase consisted of H_2_O with TFA (0.0025%) and CH_3_CN with a flow rate of 5 mL/min and the chromatogram was monitored at 205 and 210 nm. Thin-layer chromatography (TLC) was carried out using silica gel 60 F_254_ pre-coated aluminum plates (0.2 mm, Merck, Darmstadt, Germany). After developing with solvent systems, spots were visualized by spraying with 50% H_2_SO_4_ followed by heating.

### 2.2. Plant Material

The leaves of *Olyra latifolia* L. were collected at Akoupe in the Me Region (Akoupe, Ivory Coast) in February 2017 and were identified at the National Floristic Center of the UFHB Abidjan Ivory Coast. A voucher specimen UCJ 007583 was deposited at the Hebarium of this center.

### 2.3. Extraction and Isolation

The dried powdered leaves of *O. latifolia* (1.748 Kg, dry weight) were defatted in 19 L of petroleum ether (PE). The defatted powder was macerated with 19 L of CH_2_Cl_2_ for 48 h, followed by heating under reflux in 19 L of 80% MeOH. After evaporation of the solvents, 3.5 g of PE, 7.65 g of CH_2_Cl_2_, and 40 g of 80% MeOH extracts were obtained. The CH_2_Cl_2_ extract was subjected to vacuum liquid chromatography (VLC) over RP-C_18_, eluting with H_2_O-MeOH (8:2, 6:4, 4:6, 2:8, and 10:0) to obtain fractions A-E. Fraction B (209 mg) was further purified by semi-preparative HPLC using a gradient (10–25% CH_3_CN, in 45 min) to give compounds **1** (3 mg, *t*_R_ 13.63 min), **2** (2 mg, *t*_R_ 7.98 min), **6** (2 mg, *t*_R_ 15.85 min), and **7** (3 mg, *t*_R_ 18.57 min).

The 80% MeOH extract was suspended in H_2_O (500 mL) and subjected to chromatography on a Diaion HP-20 column (4.3 × 40 cm). Step gradient elution was conducted with MeOH-H_2_O (0%, 25%, 50%, 75%, and 100%, each 2 L), to give fractions 1–5, respectively.

Fraction 3 (13.9 g) was subjected to a VLC over RP-C_18_, eluted with MeOH-H_2_O (0%, 25%, 50%, 75%, and 100%). Fraction eluted with 50% MeOH (6.7 g) was further fractionated by VLC over silica gel using CH_2_Cl_2_-MeOH (10:0–5:5) as eluent. Fraction eluted with CH_2_Cl_2_-MeOH (8:2) was subjected to flash chromatography over RP-C_18_ to give compounds **3** (10 mg) and **5** (9 mg)**.** Fraction eluted with CH_2_Cl_2_-MeOH (7:3) was purified by flash chromatography over silica gel to obtain compound **9** (6 mg). Fraction eluted with CH_2_Cl_2_-MeOH-H_2_O (5:5) was separated by flash chromatography over silica gel to obtain compounds **4** (7 mg) and **8** (55 mg).

### 2.4. Compound Solubilization

The compounds tested in vitro, feddeiketone B (**1**), 2,3-Dihydroxy-1-(4-hydroxy-3,5-dimethoxyphenyl)-1-propanone (**2**), syringylglycerol (**3**), and DANA were solubilized in DMSO. In all experiments, final percentages of DMSO used were under 0.03%.

### 2.5. MTT Assay

Cell viability assays were performed on COS-7 cells (ATCC^®^ CRL-1651^™^), a fibroblast-like cell line derived from green African monkey kidney, and on human THP-1-derived macrophages (ATCC^®^ TIB-202^™^) harvested in 96 well plates at a cell density of 10,000 cells/well. Macrophages were obtained by differentiation of THP-1 cells using 50 nM phorbol-12-myristate-13-acetate (PMA, Sigma Aldrich Chimie, Saint Quentin Fallavier, France) for 72 h. Cells were incubated with feddeiketone B (**1**), 2,3-Dihydroxy-1-(4-hydroxy-3,5-dimethoxyphenyl)-1-propanone (**2**), syringylglycerol (**3**), or DANA (Sigma) for 2 or 3 h at a concentration range from 0.1 to 1 µM. Medium was removed and cells were incubated in obscurity for 4 h at 37 °C with a 3-(4-5-dimethylthiazol-2-yl)-2,5-diphenyltetrazolium bromide solution (MTT, Sigma, 5 mg/mL) diluted 1:6 in PBS. After incubation, medium was removed, wells washed with PBS, and 100 µL Dimethyl Sulfoxide (DMSO) (Sigma Aldrich, Darmstadt, Germany) were added to each well to solubilize formazan crystals. After 5 min agitation at room temperature, cell viability was assessed at 570 nm with Infinite F200 Pro (TECAN) hardware using the Magellan software.

### 2.6. Plasmids and Transfection Reagent

Plasmid encoding human PPCA protein was provided by Pr. Alessandra d’Azzo and has been described previously [32]. Plasmid encoding human NEU-1 was purchased from ImaGenes GmbH (Berlin, Germany). JetPEI DNA transfection reagent used for cell transfections was purchased from Polyplus transfection.

### 2.7. Cell Culture and Transfection

COS-7 cells were harvested in 4.5 g/L glucose Dulbecco’s Modified Eagle’s Medium (DMEM) supplemented with 10% heat-inactivated fetal bovine serum, 100 units/mL penicillin, 0.1 mg/mL streptomycin at 37 °C in a humidified atmosphere at 95% air, and 5% CO_2_. For sialidase assays, COS-7 cells were transiently transfected with plasmids encoding NEU-1/PPCA (1:2) using JetPEI according to the manufacturer’s protocol and all experiments were performed 48 h post-transfection. THP-1 cells were harvested in RPMI 1640 medium supplemented with 10% heat-inactivated fetal bovine serum, 100 units/mL penicillin, 0.1 mg/mL streptomycin at 37 °C in a humidified atmosphere at 95% air, and 5% CO_2_. THP-1 monocytes were differentiated into adherent macrophages using 50 nM PMA for 72 h.

### 2.8. Sialidase Activity

Transfected COS-7 cells harvested in 10 cm Petri dishes were washed with cold PBS and resuspended in 1 mL cold TEM buffer (75 mM Tris, 2 mM EDTA, 12 mM MgCl_2_ with a protease inhibitor cocktail, 10 mM NaF, 2 mM Na_3_VO_4_, pH 7.5). After sonication, samples were centrifuged at 600× *g* (10 min, 4 °C) to remove nuclei and non-lysed cells. Thereafter, samples were centrifuged at 20,000× *g* (45 min, 4 °C) and crude membrane-containing pellets were resuspended in 400 µL MES buffer (2-(N-Morpholino) ethanesulfonic acid hydrate, 20 mM, pH 4.5) (Sigma). After quantification of proteins with BCA protein assay (Interchim, Montluçon, France), sialidase activity at the plasma membrane was measured from 50 µg of crude membrane proteins. Vegetal compounds and DANA were incubated with crude membrane preparations for 15- or 30-min at 4 °C. Crude membrane proteins in MES buffer were then incubated with 2′-(4-Methylumbelliferyl)-alpha-ᴅ*-N*-acetylneuraminic acid (Muf-NANA, BioSynth, Staad, Switzerland) at a concentration of 400 µM for 2 h at 37 °C in obscurity. Reaction was stopped by adding Na_2_CO_3_ (Merck). Samples were then deposited in black 96 well plates and emitted fluorescence was measured with Infinite F200 Pro (TECAN, Männedorf, Switzerland) hardware and Magellan software (excitation: 360 nm/emission: 465 nm).

Sialidase activity at the plasma membrane of macrophages was performed as described previously [33]. k-elastin (kE) harboring the GxxPG bioactive motif was produced by chemical hydrolysis of insoluble elastin coming from bovine neck ligaments. Differentiated THP-1 cells, seeded in 12-well culture dishes (5 × 10^5^ cells/well), were washed with PBS and pre-incubated 15 or 30 min with vegetal compounds or DANA, a reaction buffer containing 20 mM of CH_3_COONa (pH = 6.5) and 400 µM of Muf-NANA, with or without k-elastin (50 µg/mL). After the pre-incubation step, cells were put 2 h 30 min at 37 °C in the dark. After incubation, the reaction was stopped by adding 0.4 M of glycine buffer (pH = 10.4) and the fluorescent 4-methylumbeliferone product released in the medium was measured using the Infinite F200 Pro (TECAN) hardware and Magellan software.

### 2.9. Western Blot

Protein samples in appropriate buffers according to the experiments were diluted in Laemmli buffer (62.5 mM Tris, 2% SDS, 10% glycerol, 0.05% bromophenol blue, pH 6.8) and heated 10 min at 100 °C. After electrophoresis in a 10% acrylamide SDS-PAGE gel, proteins were transferred onto a nitrocellulose membrane at 100 V for 1 h in a Tris/glycine buffer supplemented with 10% ethanol. After blocking of the nitrocellulose membrane with 0.05% TBS Tween-20 (TBS-T) supplemented with 5% milk for 1 h at room temperature, membrane was probed with primary antibodies diluted at 1:200 for NEU-1 (NEU-1 F8 Santa Cruz) and at 1:750 for β actin (Santa Cruz Biotechnology, Heidelberg, Germany) in TBS-T with 3% BSA overnight at 4 °C. Membrane was then washed in TBS-T and incubated with HRP-linked secondary antibodies diluted at 1/10,000 in TBS-T with 5% milk at room temperature. Anti-mouse HRP-linked antibodies (Cell Signaling, Danvers, MA, USA) were used for protein detections. Chemiluminescent protein detection was done using ECL Prime and ODYSSEY Fc (LI-COR Biosciences-GmbH, Bad Homburg, Germany) hardware and the Image Studio software.

### 2.10. Statistical Analysis

Results are expressed as mean ±SEM. Statistical significance was evaluated using Student *t*-test or ANOVA followed by a Dunnett’s multiple comparison test.

## 3. Results

### 3.1. Structure Identification

**Figure 1:**
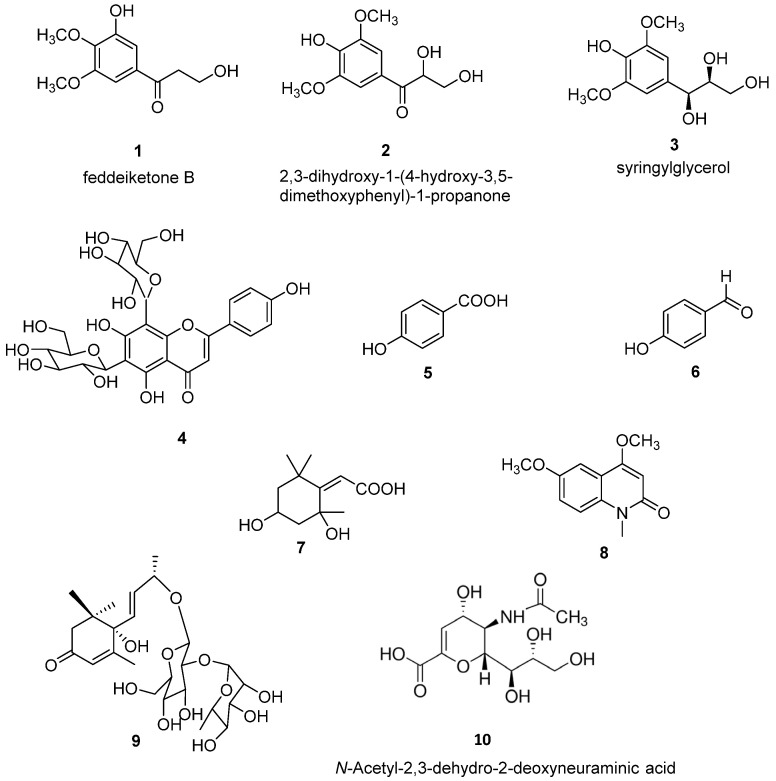
Chemical structures of compounds (**1**–**9**) isolated from *Olyra latifolia* and of *N*-Acetyl-2,3-dehydro-2-deoxyneuraminic acid (DANA) (**10**).

The purification of CH_2_Cl_2_ and 80% MeOH extracts obtained on defatted *O. latifolia* leaves gave nine compounds. Compounds 1–9 (Figure 1) were identified as feddeiketone B (**1**) [34], 2,3-dihydroxy-1-(4-hydroxy-3,5-dimethoxyphenyl)-1-propanone (**2**) [35], syringylglycerol (**3**) [36], vicenine 2 (**4**) [37], *p*-hydroxybenzoïc acid (**5**) [38], *p*-hydroxybenzaldéhyde (**6**) [39], 2,4-dihydroxy-2,6,6-triméthylcyclohexylidene acetic acid (**7**) [40], 4,6-dimethoxy-1-methylquinolin-2-(1H)-one (**8**) [41], and zizyvoside I (**9**) [42] by spectroscopic methods including 1D- and 2D-NMR experiments (1H, ^13^C, HSQC, HMBC, COSY, NOESY) in combination with HR-ESI-MS, in addition to comparison with literature data. As NEU-1 activity is implicated in the development of diabetes and as the leaves of *O. latifolia* are used to treat several pathologies such as diabetes, feddeiketone B (**1**), 2,3-Dihydroxy-1-(4-hydroxy-3,5-dimethoxyphenyl)-1-propanone (**2**) and syringylglycerol (**3**) have been selected for this study due to their structural analogy with DANA. DANA chemical structure is shown for comparison as compound 10 (Figure 1). Compounds 1–3 are phenylpropanoids in C_6_-C_3_ with a hydroxylated lateral chain in beta position of a hydroxyl group in the cycle, like in DANA. Compound **2** and syringylglycerol (**3**) have a syringyl group and a glycerol lateral chain which was oxydated in carboxyl in position 1 in compound **2**. The feddeiketone B (**1**) was slightly different with no hydroxyl in position 2 of the lateral chain (Figure 1). Even if there are no dihydropyranol group as core and no -NH-CO- in compounds **1**–**3** while they are found in DANA, several structural similarities between DANA and these three molecules have been identified: indeed, the presence of a six atom ring substituted by a 3 carbon hydroxyl chain and a hydroxyl on the beta ring of the side chain are the main structural analogies which lead us to select these molecules extracted from Olyra latifolia.

#### 3.1.1. Compound **1**

*White amorphous powder*; C_11_H_14_O_5_; EI-MS *m/z*: 249.0739 [M+Na]^+^; ^1^H-NMR (500 MHz, CD_3_OD, *δ*/ppm, *J*/Hz): 7.05 (H, d, *J* = 1.9, H-2), 6.82 (H, d, *J* = 1.9, H-6), 3.44 (1H, t, *J* = 6.2 Hz, H-8), 3.43 (2H, t, *J* = 6.2 Hz, H-9), 3.74 (3H, s, 3-O-CH_3_), 3.70 (3H, s, 4-O-CH_3_); ^13^C-NMR (125 MHz, MeOD, *δ*/ppm): 130.4 (C-1), 111.4 (C-2), 149.0 (C-3), 138.4 (C-4), 152.7 (C-5), 106.0 (C-6), 198.1 (C-7), 43.8 (C-8), 58.6 (C-9), 55.7 (3-OCH_3_), 60.7 (4-O CH_3_). The structure was determined from above data according to literature [34] and identified as feddeiketone B.

#### 3.1.2. Compound **2**

*White amorphous powder*; C_11_H_14_O_6_; EI-MS *m/z*: 265.0684 [M+Na]^+^; ^1^H-NMR (500 MHz, CD_3_OD *δ*/ppm, *J*/Hz): 7.38 (2H, s, H-2, H-6), 5.15 (1H, dd, *J* = 5.4, 3.4 Hz, H-8), 3.99 (1H, dd, *J* = 11.6, 4.5 Hz, H-9a), 3.70 (1H, dd, *J* = 11.6, 5.4 Hz, H-9a); 3.95 (6H, s, 3-OCH_3_, 5-OCH_3_); ^13^C-NMR (500 MHz, CD_3_OD, *δ*/ppm): 124.0 (C-1), 106.0 (C-2, C-6), 148.0 (C-3, C-5), 142.0 (C-4), 198.0 (C-7), 74.0 (C-8), 65.0 (C-9), 55.5 (3-OCH_3_, 5-OCH_3_). The structure was determined from the above data according to literature [35] and identified as 2,3-dihydroxy-1-(4-hydroxy-3,5-dimethoxyphenyl)-1-propanone.

#### 3.1.3. Compound **3**

*White amorphous powder*; C_11_H_14_O_6_; EI-MS *m/z*: 265.0684 [M+Na]^+^; ^1^H-NMR (500 MHz, CD_3_OD, *δ*/ppm, *J*/Hz): 6.58 (2H, s, H-2 et H-6), 4.95 (1H, d, *J* = 5.5 Hz, H-7), 3.70 (1H, m, H-8), 3.40 (1H, m, H-9a), 3.55 (1H, m, H-9b), 3.74 (6H, s, 3-OCH_3_, 5-OCH_3_); ^13^C-NMR (500 MHz, CD_3_OD, *δ*/ppm): 134.1 (C-1), 104.1 (C-2, C-6), 147.4 (C-3, C-5), 133.4 (C-4), 72.9 (C-7), 75.8 (C-8), 62.5 (C-9), 55.8 (3-OCH_3_, 5-OCH_3_). The structure was determined from the above data according to literature [36] and identified as syringylglycerol.

#### 3.1.4. Compound **4**

*Yellowish amorphous powder*; C_25_H_40_O_12_; EI-MS *m/z*: 617.1491 [M+Na]^+^; ^1^H-NMR (500 MHz, CD_3_OD, *δ*/ppm, *J*/Hz): 6.70 (1H, s, H-3), 8.00 (2H, d, *J* = 8.4 Hz, H-2′, H-6′), 6.95 (2H, d, *J* = 8.4, Hz H-3′, H-5′), 6-*C*-glc: 4.88 (1H, d, *J* = 9.7 Hz, H-1), 3.70 (1H, t, *J* = 8.9 Hz, H-2), 3.60 (1H, t, *J* = 9.7 Hz, H-3), 3.63 (1H, t, *J* = 9.4 Hz, H-4), 3.51 (1H, m, H-5), 3.84 (1H, dd, *J* = 11.7, 4.5 Hz, H-6a), 3.80 (1H, *J* = 11.7, 2.4 Hz, H-6b), 8-*C*-glc: 4.75 (1H, d, *J* = 9.7 Hz, H-1), 4.15 (1H, t, *J* = 9.4 Hz, H-2), 3.51 (1H, *J* = 9.3 Hz, H-3), 3.70 (1H, *J* = 9.1 Hz, H-4), 3.35 (1H, m, H-5), 3.91 (1H, dd, *J* = 12.1, 5.4 Hz, H-6a), 3.78 (1H, dd, *J* = 12.1, 2.1 Hz, H-6b); ^13^C-NMR (500 MHz, CD_3_OD, *δ*/ppm): 165.6 (C-2), 102.2 (C-3), 183.0 (C-4), 161.8 (C-5), 109.6 (C-6), 163.3 (C-7), 102.9 (C-8), 156.2 (C-9), 105.6 (C-10), 122.0 (C-1′), 128.0 (C-2′, C-6′), 115.8 (C-3′, C-5′), 161.0 (C-6′), 6-*C*-glc: 73.0 (C-1), 72.2 (C-2), 77.6 (C-3), 69.8 (C-4), 81.0 (C-5), 60.0 (C-6), 8-*C*-glc: 74.0 (C-1), 72.2 (C-2), 78.2 (C-3), 71.0 (C-4), 81.6 (C-5), 61.5 (C-6). The structure was determined from the above data according to literature [37] and identified as vicenine 2.

#### 3.1.5. Compound **5**

*White amorphous powder*; C_7_H_6_O_3_; EI-MS *m/z*: 161.1105 [M+Na]^+^; ^1^H-NMR (500 MHz, CD_3_OD, *δ*/ppm, *J*/Hz): 7.9 (2H, d, *J* = 8.8 Hz, H-2, H-6), 6.8 (2H, d, *J* = 8.8 Hz, H-3, H-5); ^13^C-NMR (500 MHz, CD_3_OD, *δ*/ppm): 121.5 (C-1), 131.6 (C-2, C-6), 114.6 (C-3, C-5), 161.9 (C-4), 168.9 (C-7). The structure was determined from the above data according to literature [38] and identified as *p*-hydroxybenzoïc acid.

#### 3.1.6. Compound **6**

*White amorphous powder*; C_7_H_6_O_2_; EI-MS *m/z*: 145.1111 [M+Na]^+^; ^1^H-NMR (500 MHz, CD_3_OD, *δ*/ppm, *J*/Hz): 7.90 (2H, d, *J* = 8.8 Hz, H-2, H-6), 6.85 (2H, d, *J* = 8.8 Hz, H-3, H-5), 9.80 (1H, s, H-7); ^13^C-NMR (500 MHz, CD_3_OD, *δ*/ppm): 128.0 (C-1), 132.0 (C-2, C-6), 115.0 (C-3, C-5), 163.5 (C-4), 191.0 (C-7). The structure was determined from the above data according to literature [39] and identified as *p*-hydroxybenzaldehyde.

#### 3.1.7. Compound **7**

*White amorphous powder*; C_11_H_18_O_4_; 237.1096 [M+Na]^+^; ^1^H-NMR (500 MHz, CD_3_OD, *δ*/ppm, *J*/Hz): 2.48 (1H, dt, *J* = 13.6, 2.7 Hz, H-3a), 1.75 (1H, dd, *J* = 13.6, 4.0 Hz, H-3b), 4.23 (1H, q, *J* = 3.5 Hz, H-4), 2.10 (1H, dt, *J* = 14.4, 2.7 Hz, H-5a), 1.5 (1H, dd, *J* = 14.4, 3.7 Hz, H-5b), 5.77 (1H, s, H-7), 1.76 (3H, s, 2-CH_3_), 1.49 (3H, s, 6a-CH_3_), 1.3 (3H, s, 6-CH_3_); ^13^C-NMR (500 MHz, CD_3_OD, *δ*/ppm): 184.3 (C-1), 87.6 (C-2), 45.0 (C-3), 65.8 (C-4), 46.6 (C-5), 35.8 (C-6), 111.9 (C-7), 173.0 (C-8), 26.0 (2-Me), 25.6 (6a-CH_3_), 29.6 (6-CH_3_). The structure was determined from the above data according to literature [40] and identified as 2,4-dihydroxy-2,6,6-trimethylcyclohexylidene acetic acid.

#### 3.1.8. Compound **8**

*White amorphous powder*; C_12_H_13_NO_3_; 219.0888 [M+Na]^+^; ^1^H-NMR (500 MHz, CD_3_OD, *δ*/ppm, *J*/Hz): 6.04 (1H, s, H-3), 7.40 (1H, s, H-5), 7.18 (1H, dd, *J* = 9.0, 2.8 Hz, H-7), 7.27 (1H, d, *J* = 9.0, H-8), 3.66 (3H, s, N-CH_3_), 4.86 (3H, s, 4-OCH_3_), 3.94 (3H, s, 6-OCH_3_); ^13^C-NMR (500 MHz, CD_3_OD, *δ*/ppm): 158.9 (C-2), 90.4 (C-3), 164.8 (C-4), 119.9 (C-4a), 113.4 (C-5), 150.0 (C-6), 114.9 (C-7), 125.9 (C-8), 129.9 (C-8a), 39.9 (N-CH_3_), 55.8 (4-OCH_3_), 55.6 (6-OCH_3_). The structure was determined from the above data according to literature [41] and identified as 4,6-dimethoxy-1-methylquinolin-2-(1H)-one.

#### 3.1.9. Compound **9**

*White amorphous powder*; C_25_H_40_O_12_; 555.2427 [M+Na]^+^; ^1^H-NMR (500 MHz, CD_3_OD, *δ*/ppm, *J*/Hz): 5.90 (1H, s, H-2), 2.55 (1H, d, *J* = 17.0 Hz, H-6a), 2.18 (1H, d, *J* = 17.0 Hz, H-6a), 5.88 (1H, d, *J* = 16.0 Hz, H-7), 5.86 (1H, dd, *J* = 16.0, 7.0 Hz, H-8), 4.42 (1H, dq, J = 16.0, 7 Hz, H-9), 1.32 (1H, d, *J* = 6.4 Hz, H-10), 1.94 (3H, s, H-11), 1.20 (3H, s, 12-CH_3_), 1.26 (3H, s, 13-CH_3_), Glc: 4.45 (1H, d, *J* = 7.8 Hz, H-1), 3.43 (1H, t, *J* = 8.8 Hz, H-2), 3.47 (1H, t, *J* = 8.8 Hz, H-3), 3.22 (1H, t, *J* = 8.9 Hz, H-4), 3.25 (1H, m, H-5), 3.87 (1H, dd, *J* = 12.0, 4.7 Hz, H-6a), 3.63 (1H, dd, *J* = 12.0, 2.1 Hz, H-6b), Rha: 5.26 (1H, d, 1.2 Hz, H-1), 3.92 (1H, dd, *J* = 3.3, 1.2 Hz, H-2), 3.67 (1H, dd, *J* = 9.5, 3.3 Hz, H-3), 3.38 (1H, t, *J* = 9.5 Hz, H-4), 4.10 (1H, m, H-5), 1.27 (1H, d, *J* = 6.2 Hz, H-6); ^13^C-NMR (500 MHz, CD_3_OD, *δ*/ppm): 199.8 (C-1), 125.8 (C-2), 165.8 (C-3), 78.1 (C-4), 41.1 (C-5), 49.3 (C-6), 130.2 (C-7), 133.9 (C-8), 75.8 (C-9), 19.8 (C-10), 18.2 (C-11), 23.3 (12-CH_3_), 22.0 (13-CH_3_), Glc: 99.8 (C-1), 77.3 (C-2), 78.0 (C-3), 70.5 (C-4), 76.6 (C-5), 61.5 (C-6), Rha: 100.6 (C-1), 70.8 (C-2), 70.9 (C-3), 72.6 (C-4), 68.3 (C-5), 16.7 (C-6). The structure was determined from the above data according to literature [42] and identified as zizyvoside I.

### 3.2. Effects of Selected Compounds **1**–**3** on Cell Viability

**Figure 2:**
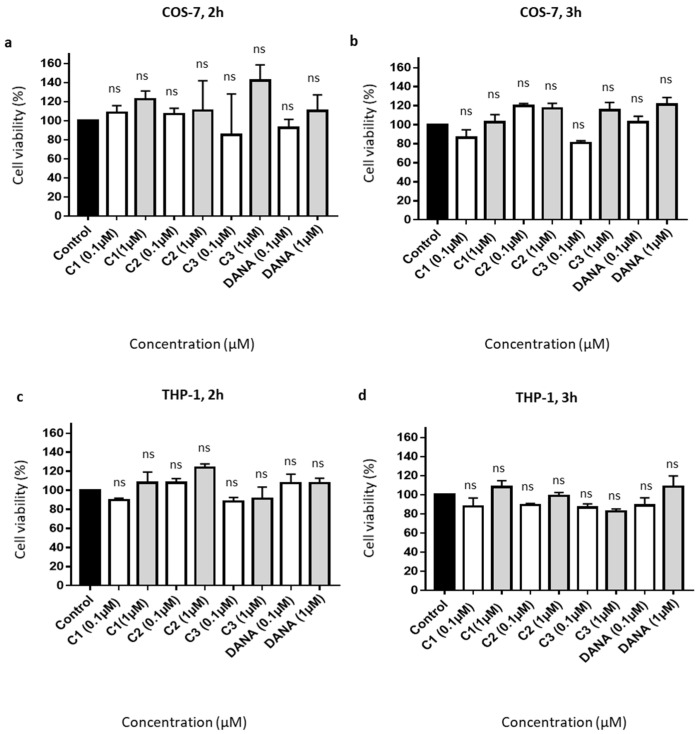
Effects of the vegetal compounds and of N-Acetyl-2,3-dehydro-2-deoxyneuraminic acid on cell viability. (**a**–**d**) 10,000 COS-7 and THP-1 cells were plated in 96 well plates. Cells were incubated with the natural compounds 1, 2, 3, or DANA at 0.1 or 1 µM. (**a**) Effects on COS-7 cell viability after 2 h. (**b**) Effects on COS-7 cell viability after 3 h. (**c**) Effects on THP-1 cell viability after 2 h. (**d**) Effects on THP-1 cell viability after 3 h. Results were normalized to the control condition expressed at 100%. (*n* = 3–4), (ANOVA). Each run in triplicates, (C1: feddeiketone B, C2: 2,3-Dihydroxy-1-(4-hydroxy-3,5-dimethoxyphenyl)-1-propanone, C3: syringylglycerol, DANA: N-Acetyl-2,3-dehydro-2-deoxyneuraminic acid), (ns: non-significant).

Effects on cell viability of feddeiketone B (compound **1**), 2,3-dihydroxy-1-(4-hydroxy-3,5-dimethoxyphenyl)-1-propanone (compound **2**), syringylglycerol (compound **3**), and the reference DANA molecule were evaluated in COS-7 and THP-1 cell lines. For all these compounds, no cell toxicity was noticed both in COS-7 (Figure 2a,b) and THP-1 (Figure 2c,d) cell lines after 2- or 3-h incubation at 0.1 and 1 µM in comparison with control (untreated cells). Together, these results show that DANA and compounds **1**–**3** have no significant effects on cell viability.

### 3.3. Effects of Selected Compounds **1**–**3** on Sialidase Activity in COS-7 Cells Overexpressing NEU-1

**Figure 3:**
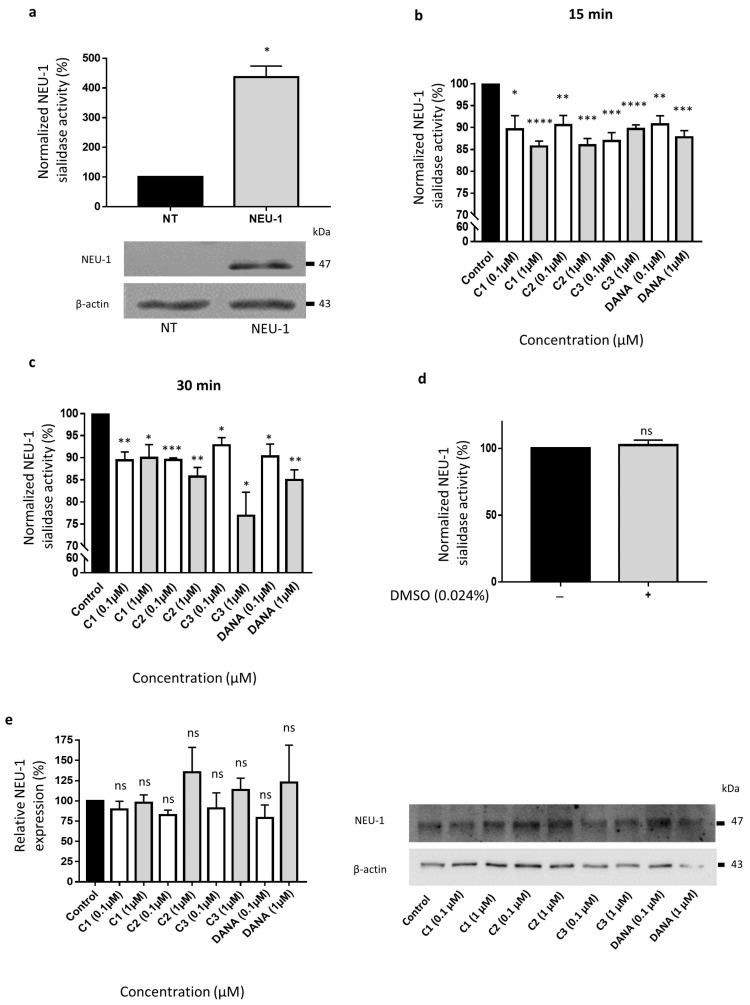
Effects of N-Acetyl-2,3-dehydro-2-deoxyneuraminic acid and compounds **1**–**3** on membrane NEU-1 sialidase activity in NEU-1 overexpressing cells. (**a**) Sialidase activities in untransfected (NT) and transfected (NEU-1) COS-7 cells. The bottom panel indicates the expression of NEU-1 in untransfected (NT) and transfected (NEU-1) COS-7 cells. β-actin was used as an internal control (*n* = 3) (*t* test). (**b**) Normalized sialidase activity after 15 min incubation with the vegetal compounds at 0.1 or 1 µM and comparison with DANA (*n* = 5–6) (*t* test). (**c**) Normalized sialidase activity after 30 min incubation with DANA and the compounds **1**–**3** at 0.1 or 1 µM. Results are represented compared to the nothing condition normalized to 100% (*n* = 3–6) (*t* test). (**d**) Effects of DMSO alone on NEU-1 sialidase activity (*n* = 7) (*t* test). (**e**) Relative NEU-1 expression in presence of the vegetal compounds and DANA at 0.1 and 1 µM. The expression of NEU-1 was normalized to β-actin (*n* = 3), (ANOVA), (C1: feddeiketone B, C2: 2,3-Dihydroxy-1-(4-hydroxy-3,5-dimethoxyphenyl)-1-propanone, C3: syringylglycerol, DANA: N-Acetyl-2,3-dehydro-2-deoxyneuraminic acid), (ns: non-significant, * *p* < 0.05, ** *p* < 0.01, *** *p* < 0.001, **** *p* < 0.0001).

We next evaluated the ability of compounds **1**–**3** to decrease membrane sialidase activity of COS-7 cells overexpressing NEU-1. COS-7 cells were transfected with both human NEU-1 and PPCA encoding plasmids, and the capacity of the compounds **1**–**3** to inhibit sialidase activity was assessed 48 h post-transfection in crude membrane preparations and compared to DANA at the same concentrations. A four-fold increase of membrane sialidase activity, from 100 to 437 ± 37%, was observed between untransfected cells and COS-7 cells overexpressing NEU-1 (Figure 3a). After 15 min of incubation at 0.1 and 1 µM, both DANA and the vegetal compounds are able to decrease NEU-1 sialidase activity in a similar way (Figure 3b). Compounds **1**–**3** and DANA at 0.1 µM decrease NEU-1 sialidase activity by 10.4 ± 3.1, 9.4 ± 2.1, 13.1 ± 1.8, and 9.3 ± 1.9%, respectively, in comparison with the control (untreated cells) (Figure 3b). A treatment by compounds **1**–**3** and DANA at 1 µM decreases NEU-1 sialidase activity by 14.3 ± 1.3, 14.0 ± 1.5, 10.3 ± 0.9, and 12.2 ± 1.5%, respectively, in comparison with the control (Figure 3b). Similar effects were observed after 30 min of incubation at both 0.1 and 1 µM (Figure 3c). Given that all the experiments were performed in presence of 0.024% of DMSO (final concentration), effects of DMSO alone at the same concentration were evaluated. No effect of DMSO was observed compared to the control condition (untreated cells) (Figure 3d). Moreover, DANA and the vegetal compounds do not alter significantly NEU-1 membrane expression level in transfected COS-7 cells (Figure 3e). Altogether, these results suggest that these three vegetal compounds are able to decrease significantly NEU-1 mediated sialidase activity in an overexpression cell model, in similar extent to the DANA molecule.

### 3.4. Effects of Compounds **1**–**3** on Membrane NEU-1 Mediated Sialidase Activity Triggered by Elastin-Derived Peptides in THP-1 Cells

**Figure 4:**
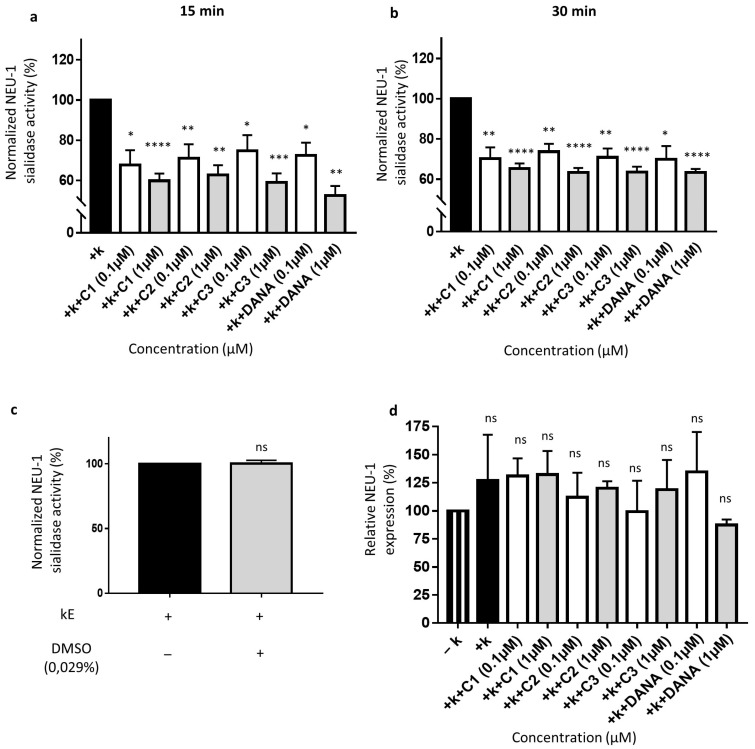
Effects of N-Acetyl-2,3-dehydro-2-deoxyneuraminic acid and compounds **1**–**3** on membrane NEU-1 sialidase activity in full adherent macrophages. Adherent macrophages were incubated with DANA and the vegetal compounds and sialidase activity was triggered or not by k-elastin. (**a**) Normalized membrane NEU-1 sialidase activity after 15 min incubation with DANA or compounds **1**–**3** at 0.1 or 1 µM under k-elastin stimulation (*n* = 3–6) (*t* test). (**b**) Normalized membrane NEU-1 sialidase activity after 30 min incubation with DANA or compounds **1**–**3** at 0.1 or 1 µM under k-elastin stimulation.%. (*n* = 3–8) (*t* test). Results are represented compared to the control condition (+ k-elastin) normalized to 100%. (**c**) Effects of DMSO alone on membrane NEU-1 sialidase activity under k-elastin stimulation (*n* = 4) (*t* test). (**d**) Relative NEU-1 expression in presence of the vegetal compounds and DANA at 0.1 and 1 µM. The expression of NEU-1 was normalized to β-actin (*n* = 3), (ANOVA) (C1: feddeiketone B, C2: 2,3-Dihydroxy-1-(4-hydroxy-3,5-dimethoxyphenyl)-1-propanone, C3: syringylglycerol, DANA: N-Acetyl-2,3-dehydro-2-deoxyneuraminic acid), (ns: non-significant, * *p* < 0.05, ** *p* < 0.01, *** *p* < 0.001, **** *p* < 0.0001).

As previously reported, membrane sialidase activity triggered by k-elastin stimulation of THP-1-derived macrophages is dependent of NEU-1 [33]. After k-elastin stimulation (50 µg/mL), NEU-1 sialidase activity of macrophages increases by 54.1 ± 9.0% compared to non-stimulated cells for an incubation period of 15 min and by 51.5 ± 26.9% for an incubation period of 30 min (not shown). k-elastin stimulated sialidase activity decreases in presence of DANA and the vegetal compounds after 15 min of incubation at 0.1 µM. Indeed, in presence of compounds **1**–**3** and DANA, decreases of k-elastin induced NEU-1 sialidase activity of 32.3 ± 7.3, 28.9 ± 6.9, 25.2 ± 7.7, and 27.7 ± 6.4% are observed, respectively (Figure 4a). These inhibitory effects were increased at a concentration of 1 µM showing inhibitions of 40.2 ± 3.6, 37.2 ± 4.8, 41.0 ± 4.5, and 47.5 ± 7.34.7% for compounds **1**–**3** and DANA, respectively (Figure 4a). After 30 min in presence of compounds **1**–**3** and DANA at 0.1 µM, decreases of k-elastin induced NEU-1 sialidase activity of 29.7 ± 5.4, 26.3 ± 3.9, 29.2 ± 4.4, and 30.2 ± 6.7% were observed, respectively (Figure 4b). After 30 min in presence of compounds **1**–**3** and DANA at 1 µM, decreases of k-elastin induced NEU-1 sialidase activity of 34.7 ± 2.5, 36.6 ± 2.2, 36.5 ± 2.7, and 36.7 ± 1.7% were observed, respectively (Figure 4b). As for COS-7 cells, DMSO alone (0.029%) did not affect the sialidase activity in THP-1-derived macrophages (Figure 4c). Furthermore, as for COS-7 cells, DANA and the vegetal compounds do not alter NEU-1 expression in THP-1-derived macrophages with or without k-elastin stimulation (Figure 4d). Taken together, these results show that the natural compounds **1**–**3** strongly inhibit NEU-1 sialidase activity triggered by k-elastin, in similar extent to the DANA molecule.

In both cell lines, compounds **1**–**3** act quickly at low concentrations to inhibit NEU-1 sialidase activity. These inhibitory effects of vegetal compounds were more important in THP-1-derived macrophages under k-elastin stimulation than in COS-7 cells overexpressing NEU-1. All vegetal compounds tested show comparable sialidase activity inhibition ranges. Their inhibition potentials were also similar to those observed with DANA at same concentrations.

## 4. Discussion

The key role of NEU-1 in the degradation of sialyloconjugates in lysosomes has been known for many years [43], but its implication in cellular regulatory mechanisms has been identified only recently. These new findings are associated with the discovery of NEU-1 localization at the cell plasma membrane. As described previously, human NEU-1, the ERC catalytic subunit, is known, after activation of its sialidase activity by EDP, to be involved in several pathophysiological contexts as cancers [7,8,9,10,11,13,14,44], diabetes [3], atherosclerosis [4], and nonalcoholic steatohepatitis [6]. That is why several strategies have been envisaged to disturb the activation of NEU-1 to prevent the progression of various diseases. Among these, chemical compounds [22,45] or transmembrane peptides as inhibitors of NEU-1 dimerization/activation [24] are currently under development. During the last years, different selective inhibitors of human NEU-1 have been notably developed based on the DANA chemical structure [22,23]. Indeed, the C9-amido analog of DANA (C9-BA-DANA) shows micromolar IC50 against human NEU-1 [23] and the C5-hexanamido-C9-acetamido analog has a *K_i_* of 53 ± 5 nM and 340-fold selectivity over other isoenzymes [22]. In fact, C9-BA-DANA not only blocks NEU-1 sialidase activity but also its bioactivities in human lung in vitro and murine lung in vivo [45]. Furthermore, a recent study has also shown that interfering peptides targeting the transmembrane domain of NEU-1 are able to decrease both NEU-1 dimerization and sialidase activity [24]. The aim of this study was to identify plant-derived natural products that share analogy with the DANA molecule and to evaluate their bioactivity against plasma membrane NEU-1 sialidase activity. We have selected *Olyra latifolia* because in Ivory Coast, their leaves were used for treating diabetes and the diabetic wound [28] and this activity could be related to NEU-1 inhibitory activity [3]. Nine known compounds were isolated from the leaves of *Olyra latifolia* and three compounds (**1**–**3**) were shown to possess structural analogy with DANA: the feddeiketone B, the 2,3-dihydroxy-1-(4-hydroxy-3,5-dimethoxyphenyl)-1-propanone, and the syringylglycerol. Two cell models have been used in the present work, COS-7 cells which overexpressed NEU-1 and THP-1-derived macrophages which endogenously express this target protein. For all these compounds and DANA, no significant effect on cell toxicity was observed in the two cell lines after 2 or 3 h of incubation at 0.1 and 1 µM. Therefore, we studied the effects of these compounds on NEU-1 sialidase activity in COS-7 cells and in THP-1-derived macrophages at this concentration range. In all experiments, final percentages of DMSO used were under 0.03% in order to avoid cell toxicity. Our results suggest that compounds **1**–**3** are able to decrease significantly NEU-1 mediated sialidase activity in both cell models, but the inhibitory effects were much greater in THP-1 cells than in COS-7 for all the tested concentrations (0.1 and 1 µM). These results might be explained by the fact that in COS-7 cells, NEU-1 is overexpressed and, therefore, the ratio between the NEU-1 expression level and the compound concentrations is higher in these cells compared to THP-1 cells. Kawecki et al. have previously shown that incubation with k-elastin significantly increased sialidase activity at the plasma membrane and that silencing NEU-1 by siRNA completely blocked the effects of k-elastin: these data definitely demonstrated involvement of NEU1 in k-elastin-induced sialidase activity at the plasma membrane of THP-1 cells [33]. Our results obtained with the same cell model showed that the natural compounds 1, 2 and 3 strongly inhibit NEU-1 sialidase activity triggered by k-elastin: these experiments suggest that these 3 compounds are able to directly inhibit human NEU-1, the ERC catalytic subunit, after activation of its sialidase activity by k-elastin. In addition to these results, we observed that the inhibitory effects of the three tested compounds extracted from *Olyra latifolia* are very similar to those obtained with DANA in the same experimental conditions which indicate that these natural molecules are potent inhibitor of NEU-1 as well as DANA. The glycerol lateral chain in syringylglycerol (**3**) and DANA seems to be important for the activity or at least a ketone and a primary alcohol group on a C_3_-substituant. In this preliminary study, we tested the effects of these three compounds at very low concentrations (0.1 and 1 µM). Indeed, these compounds are solubilized in DMSO which cannot be used at higher concentrations due to its toxicity. However, the effects of these molecules obtained at low concentrations are very promising and suggest that at higher concentrations (which requires less toxic solvents for solubilization), similar to those classically used for DANA, these inhibitory effects could be more important. Consequently, these vegetal compounds could be used as natural putative inhibitors of NEU-1 and could serve as models to design more potent synthetic inhibitors. Furthermore, as these compounds appear to be effective in inhibiting membrane NEU-1 in vitro, they could be used in in vivo models to inhibit this protein involved in several diseases such as atherosclerosis, thrombosis, insulin resistance, non-alcoholic steatohepatitis, and cancer.

In this preliminary study, we report the isolation and characterization of nine compounds (**1**–**9**) isolated for the first time from *O. latifolia* leaves. Among them, compounds **1**–**3**—having structural analogy with DANA—showed inhibitory effects against human NEU-1-mediated sialidase activity at the plasma membrane. The effects of these molecules obtained at low concentrations are very promising and suggest that at higher concentrations, similar to those classically used for DANA, these inhibitory effects could be more important. Thus, these compounds are considered as new natural bioactive inhibitors of human NEU-1 which may be further exploited as potential leads to elucidate the biological functions of this sialidase and to target this protein involved in several pathophysiological contexts. Consequently, this preliminary study predicted an inhibitory potential of these vegetal compounds that could be a starting point for the development of new natural putative inhibitors of NEU-1 and for the design of more potent synthetic inhibitors (identified by determining their IC50 and *K_i_* values and comparing with those of other inhibitors). Furthermore, in order to better characterize compounds **1**–**3** and their possible use as tools for a better understanding of sialic acid biology, it could be interesting to evaluate their effect on another plasma membrane associated sialidase such as NEU-3.but also on two other members of sialidase family, NEU-2 and NEU-4. Last, as these compounds appear to be effective in inhibiting membrane NEU-1 in vitro, they could be used in in vivo models to down-regulate this protein involved in several diseases such as atherosclerosis, thrombosis, insulin resistance, non-alcoholic steatohepatitis, and cancer.

## Data Availability

Data is contained within the article.

## References

[1] Giacopuzzi E., Bresciani R., Schauer R., Monti E., Borsani G. (2012). New Insights on the Sialidase Protein Family Revealed by a Phylogenetic Analysis in Metazoa. PLoS ONE.

[2] Bennasroune A., Romier-Crouzet B., Blaise S., Laffargue M., Efremov R.G., Martiny L., Maurice P., Duca L. (2019). Elastic Fibers and Elastin Receptor Complex: Neuraminidase-1 Takes the Center Stage. Matrix Biol..

[3] Blaise S., Romier B., Kawecki C., Ghirardi M., Rabenoelina F., Baud S., Duca L., Maurice P., Heinz A., Schmelzer C.E.H. (2013). Elastin-Derived Peptides Are New Regulators of Insulin Resistance Development in Mice. Diabetes.

[4] Gayral S., Garnotel R., Castaing-Berthou A., Blaise S., Fougerat A., Berge E., Montheil A., Malet N., Wymann M.P., Maurice P. (2014). Elastin-Derived Peptides Potentiate Atherosclerosis through the Immune Neu1-PI3Kγ Pathway. Cardiovasc. Res..

[5] Li J., van der Wal D.E., Zhu G., Xu M., Yougbare I., Ma L., Vadasz B., Carrim N., Grozovsky R., Ruan M. (2015). Desialylation Is a Mechanism of Fc-Independent Platelet Clearance and a Therapeutic Target in Immune Thrombocytopenia. Nat. Commun..

[6] Romier B., Ivaldi C., Sartelet H., Heinz A., Schmelzer C.E.H., Garnotel R., Guillot A., Jonquet J., Bertin E., Guéant J.-L. (2018). Production of Elastin-Derived Peptides Contributes to the Development of Nonalcoholic Steatohepatitis. Diabetes.

[7] Hornebeck W., Robinet A., Duca L., Antonicelli F., Wallach J., Bellon G. (2005). The Elastin Connection and Melanoma Progression. Anticancer Res..

[8] Ntayi C., Labrousse A.-L., Debret R., Birembaut P., Bellon G., Antonicelli F., Hornebeck W., Bernard P. (2004). Elastin-Derived Peptides Upregulate Matrix Metalloproteinase-2-Mediated Melanoma Cell Invasion through Elastin-Binding Protein. J. Investig. Dermatol..

[9] Pocza P., Süli-Vargha H., Darvas Z., Falus A. (2008). Locally Generated VGVAPG and VAPG Elastin-Derived Peptides Amplify Melanoma Invasion via the Galectin-3 Receptor. Int. J. Cancer.

[10] Haxho F., Allison S., Alghamdi F., Brodhagen L., Kuta V.E., Abdulkhalek S., Neufeld R.J., Szewczuk M.R. (2014). Oseltamivir Phosphate Monotherapy Ablates Tumor Neovascularization, Growth, and Metastasis in Mouse Model of Human Triple-Negative Breast Adenocarcinoma. Breast Cancer.

[11] Nicoloff G., Deliiyski T., Nikolov A. (2010). Detection of Serum Collagen Collagen Type IV and Elastin Derived Peptides in Patients with Breast Cancer. Diabetol. Croat..

[12] Salesse S., Odoul L., Chazée L., Garbar C., Duca L., Martiny L., Mahmoudi R., Debelle L. (2018). Elastin Molecular Aging Promotes MDA-MB-231 Breast Cancer Cell Invasiveness. FEBS Open Biol..

[13] Thulasiraman P., Kerr K., McAlister K., Hardisty S., Wistner A., McCullough I. (2019). Neuraminidase 1 Regulates Proliferation, Apoptosis and the Expression of Cadherins in Mammary Carcinoma Cells. Mol. Cell. Biochem..

[14] Ren L., Zhang L., Huang S., Zhu Y., Li W., Fang S., Shen L., Gao Y. (2016). Effects of Sialidase NEU1 SiRNA on Proliferation, Apoptosis, and Invasion in Human Ovarian Cancer. Mol. Cell. Biochem..

[15] Chavas L.M.G., Tringali C., Fusi P., Venerando B., Tettamanti G., Kato R., Monti E., Wakatsuki S. (2005). Crystal Structure of the Human Cytosolic Sialidase Neu2. Evidence for the Dynamic Nature of Substrate Recognition. J. Biol. Chem..

[16] Magesh S., Suzuki T., Miyagi T., Ishida H., Kiso M. (2006). Homology Modeling of Human Sialidase Enzymes NEU1, NEU3 and NEU4 Based on the Crystal Structure of NEU2: Hints for the Design of Selective NEU3 Inhibitors. J. Mol. Graph. Model..

[17] O’Shea L.K., Abdulkhalek S., Allison S., Neufeld R.J., Szewczuk M.R. (2014). Therapeutic Targeting of Neu1 Sialidase with Oseltamivir Phosphate (Tamiflu®) Disables Cancer Cell Survival in Human Pancreatic Cancer with Acquired Chemoresistance. Onco. Targets Ther..

[18] Scandolera A., Odoul L., Salesse S., Guillot A., Blaise S., Kawecki C., Maurice P., El Btaouri H., Romier-Crouzet B., Martiny L. (2016). The Elastin Receptor Complex: A Unique Matricellular Receptor with High Anti-Tumoral Potential. Front. Pharmacol..

[19] Duca L., Blanchevoye C., Cantarelli B., Ghoneim C., Dedieu S., Delacoux F., Hornebeck W., Hinek A., Martiny L., Debelle L. (2007). The Elastin Receptor Complex Transduces Signals through the Catalytic Activity of Its Neu-1 Subunit. J. Biol. Chem..

[20] Hinek A., Bodnaruk T.D., Bunda S., Wang Y., Liu K. (2008). Neuraminidase-1, a Subunit of the Cell Surface Elastin Receptor, Desialylates and Functionally Inactivates Adjacent Receptors Interacting with the Mitogenic Growth Factors PDGF-BB and IGF-2. Am. J. Pathol..

[21] Privitera S., Prody C.A., Callahan J.W., Hinek A. (1998). The 67-KDa Enzymatically Inactive Alternatively Spliced Variant of Beta-Galactosidase Is Identical to the Elastin/Laminin-Binding Protein. J. Biol. Chem..

[22] Guo T., Héon-Roberts R., Zou C., Zheng R., Pshezhetsky A.V., Cairo C.W. (2018). Selective Inhibitors of Human Neuraminidase 1 (NEU1). J. Med. Chem..

[23] Magesh S., Moriya S., Suzuki T., Miyagi T., Ishida H., Kiso M. (2008). Design, Synthesis, and Biological Evaluation of Human Sialidase Inhibitors. Part 1: Selective Inhibitors of Lysosomal Sialidase (NEU1). Bioorg. Med. Chem. Lett..

[24] Albrecht C., Kuznetsov A.S., Appert-Collin A. (2020). Transmembrane Peptides as a New Strategy to Inhibit Neuraminidase-1 Activation. Front. Cell Dev. Biol..

[25] Albrecht C., Appert-Collin A., Bagnard D., Blaise S., Romier-Crouzet B., Efremov R.G., Sartelet H., Duca L., Maurice P., Bennasroune A. (2020). Transmembrane Peptides as Inhibitors of Protein-Protein Interactions: An Efficient Strategy to Target Cancer Cells?. Front. Oncol..

[26] Burkill H.M. (1994). The Useful Plants of West Tropical Africa. Vol. 2: Families E-I.

[27] Vergiat A.-M. (1970). Plantes magiques et médicinales des Féticheurs de l’Oubangui (Région de Bangui) (suite). JATBA.

[28] Bamba B. (2015). Bulletin Official de La Propriété Industrielle (BIOPI). Brevets d’inventions.

[29] Coe F.G., Parikh D.M., Johnson C.A., Anderson G.J. (2012). The Good and the Bad: Alkaloid Screening and Brineshrimp Bioassays of Aqueous Extracts of 31 Medicinal Plants of Eastern Nicaragua. Pharm. Biol..

[30] DeFilipps R.A., Marina S.L., Crepin J. (2004). Medicinal Plants of the Guianas (Guyana, Surinam, French Guiana).

[31] Gibbs R.D. (1974). Chemotaxonomy of Flowering Plants.

[32] Bonten E.J., Campos Y., Zaitsev V., Nourse A., Waddell B., Lewis W., Taylor G., d’Azzo A. (2009). Heterodimerization of the Sialidase NEU1 with the Chaperone Protective Protein/Cathepsin A Prevents Its Premature Oligomerization. J. Biol. Chem..

[33] Kawecki C., Bocquet O., Schmelzer C.E.H., Heinz A., Ihling C., Wahart A., Romier B., Bennasroune A., Blaise S., Terryn C. (2019). Identification of CD36 as a New Interaction Partner of Membrane NEU1: Potential Implication in the pro-Atherogenic Effects of the Elastin Receptor Complex. Cell. Mol. Life Sci..

[34] Lu Y., Li X., Mu H., Huang H., Li G.-P., Hu Q. (2012). Bioactive Phenylpropanoids from Daphne Feddei. J. Braz. Chem. Soc..

[35] Lee T.-H., Kuo Y.-C., Wang G.-J., Kuo Y.-H., Chang C.-I., Lu C.-K., Lee C.-K. (2002). Five New Phenolics from the Roots of *Ficus b. Eecheyana*. J. Nat. Prod..

[36] Otsuka H., Takeuchi M., Inoshiri S., Sato T., Yamasaki K. (1989). Phenolic Compounds from Coix Lachryma-Jobi Var. Ma-Yuen. Phytochemistry.

[37] Velozo L.S.M., Ferreira M.J.P., Santos M.I.S., Moreira D.L., Guimarães E.F., Emerenciano V.P., Kaplan M.A.C. (2009). C-Glycosyl Flavones from Peperomia Blanda. Fitoterapia.

[38] Xin X.L., Aisa H.A., Wang H.Q. (2008). Flavonoids and Phenolic Compounds from Seeds of the Chinese Plant Nigella Glandulifera. Chem. Nat. Compd..

[39] Shimada A., Takeuchi S., Nakajima A., Tanaka S., Kawano T., Kimura Y. (2000). Phytotoxicity of Indole-3-Acetic Acid Produced by the Fungus. Pythium Aphanidermatum Biosci. Biotechnol. Biochem..

[40] Wu Y., Su J., Guo R., Ren T., Zhang M., Dong M., Sauriol F., Shi Q., Gu Y., Huo C. (2014). Two New Non-Taxoids from Leaves of Taxus Cuspidata. Chem. Nat. Compd..

[41] Campbell W.E., Davidowitz B., Jackson G.E. (1990). Quinolinone Alkaloids from an Agathosma Species. Phytochemistry.

[42] Okamura N., Yagi A., Nishioka I. (1981). Studies on the Constituents of Zizyphi Fructus. V. Structures of Glycosides of Benzyl Alcohol, Vomifoliol and Naringenin. Chem. Pharm. Bull..

[43] Bonten E.J., Annunziata I., d’Azzo A. (2014). Lysosomal Multienzyme Complex: Pros and Cons of Working Together. Cell. Mol. Life Sci..

[44] Hou G., Liu G., Yang Y., Li Y., Yuan S., Zhao L., Wu M., Liu L., Zhou W. (2016). Neuraminidase 1 (NEU1) Promotes Proliferation and Migration as a Diagnostic and Prognostic Biomarker of Hepatocellular Carcinoma. Oncotarget.

[45] Hyun S.W., Liu A., Liu Z., Cross A.S., Verceles A.C., Magesh S., Kommagalla Y., Kona C., Ando H., Luzina I.G. (2016). The NEU1-Selective Sialidase Inhibitor, C9-Butyl-Amide-DANA, Blocks Sialidase Activity and NEU1-Mediated Bioactivities in Human Lung in Vitro and Murine Lung in Vivo. Glycobiology.

